# Violence at work: determinants & prevalence among health care workers, northwest Ethiopia: an institutional based cross sectional study

**DOI:** 10.1186/s40557-019-0288-6

**Published:** 2019-04-03

**Authors:** Dawit Getachew Yenealem, Manay Kifle Woldegebriel, Ararso Tafese Olana, Tesfaye Hambisa Mekonnen

**Affiliations:** 1 Department of Environmental and Occupational Health and Safety, Institute of Public Health, College of Medicine and Health Sciences, University of Gondar, P.O. Box 196, Gondar, Ethiopia; 2 Department of Public Health, College of Health Science and Comprehensive Specialized Hospital, Aksum University, Axum, Ethiopia; 3 Department of Public Health, College of Medicine and Health Science, Ambo University, Ambo, Ethiopia

**Keywords:** Health facilities, Health care workers, Workplace violence, Gondar

## Abstract

**Background:**

Workplace violence is the intentional use of power, threatened or actual, against another person or against a group, in work-related circumstances, that either results in or has a high degree of likelihood of resulting in injury, death, psychological harm, mal development, or deprivation. The aim of this study is to assess magnitude and predictors of workplace violence among healthcare workers in health facilities of Gondar city.

**Methods:**

Institutional based cross sectional study design was employed to conduct this study. The study conducted in Gondar town from February 21 to march 21, 2016. Five hundred fifty three health care workers selected from health facilities of Gondar City administration. A stratified sampling technique was used for selecting the study subjects through simple random sampling. Data was collected by structured self administered questionnaire which is adapted from ILO/ICN/WHO/PSI after it is pretested & prepared in Amharic. The data was coded and entered in to EPI info version 7 and exported to SPSS version 20 software for analysis. The degree of association for variables was assessed using odds ratios with 95% confidence interval and *p*-value ≤0.05.

**Results:**

The prevalence of workplace violence was found to be 58.2% with [95% confidence interval (CI): (53.7, 62.3)] in which verbal abuse 282(53.1%) followed by physical attack 117(22.0%) and 38(7.2%) sexual harassment. Working at emergency departments [AOR = 3.99,95% CI:(1.49,10.73)], working at shifts [AOR = 1.98,95%, CI: (1.28,3.03)],short experiences [AOR = 3.09,95% CI: (1.20,7.98)], being nurse or midwife [AOR = 4.06, 95% CI: (1.20,13.74)] were positively associated with workplace violence. The main sources of violence are visitors/patient relatives followed by colleagues and patients.

**Conclusion:**

workplace violence is major public health problem across health facilities and the Ministry of Health should incorporate interventions in its different health sector development & management safety initiative.

## Background

Workplace violence is the intentional use of power, threatened or actual, against another person or against a group, in work-related circumstances, that either results in or has a high degree of likelihood of resulting in injury, death, psychological harm, mal development, or deprivation [1]. It has become an alarming phenomenon worldwide and one of the largest public health problem [2]. Even though workplace violence occurred on both private & public HCWs South African study shows public HCWs are more exposed than their private counterparts [3]. The rate of assaults on health workers is higher than that of other occupations-eight assaults per 10,000 workers compared with two per 10,000 for the general workplace [4].

Workplace violence causes ill health [5]. A longitudinal study on nurses demonstrates those who exposed to workplace violence develops higher somatic & musculoskeletal disorder symptoms than the comparison groups [5]. A global review of 150,000 nurses shows that third of have been physically assaulted, bullied, or injured, while around two-thirds have experienced nonphysical assault [4]. A retrospective database review among United States nurses about cost of workplace violence find annual workplace violence charges for the 2.1%of nurses reporting injuries were $94,156 ($78,924 for treatment and $15,232 for indemnity) [6].

In Ethiopia Most of healthcare workers are women in which they are exposed for both domestic & workplace violence which is a double burden [7]. Even though workplace violence studies mostly focuses on psychiatric and emergency department settings it is significantly prevalent in general healthcare workers [2].

Healthcare workers who exposed to workplace violence result to enormous consequences [8]. A systematic review of literature comes up with seven types of consequences namely: physical, psychological, emotional, work functioning, relationship with patients/quality of care, social/general, and financial. Psychological (e.g., posttraumatic stress, depression) and emotional (e.g., anger, fear) consequences and impact on work functioning (e.g., sick leave, job satisfaction) [9]. The most frequent and important effects of workplace violence as Longitudinal study from Finland shows physical violence lead to increment on intention of physician to leave while job satisfaction is affected by both bulling & physical violence [10]. In musculoskeletal studies WPV especially physical violence shows a significant role in predisposing to acute & chronic low back pain in study in Iran [11]. The consequences workplace violence extends beyond economic burden to be matter of quality of life [12].

In Ethiopia Very little information is available about workplace violence among healthcare workers. Considering large number of work force in health care sector in which majority of them are women. Currently government concern & intervention is limited domestic violence. But violence is not limited to house hold level and tackling demands comprehensive knowledge & focusing its effect on healthcare delivery as well. This study will provided a comprehensive baseline of workplace violence in healthcare workers which helps concerned bodies to initiates & start to shape strategies.

## Methods

### Study design, sample size determination, sampling technique

Institutional based quantitative cross sectional study was conducted in Gondar city in march 2016. Gondar is one of tourist destination city in Ethiopia, which is 747 km from Addis Abeba and 170 km from regional capital Bahir Dar. According to CSA 2014 projection Gondar has 323,875 populations reside in it. There are one university hospital 8 health centres. In addition 1 medium hospital, 13 specialty clinics, 15 medium clinics & 11 primary clinics according to Gondar city administration health department. About 994 health care workers employed at all level of health facilities. Sample size was determined by using single population proportion formula, taking 29.9% [13] prevalence of workplace violence from previous study in Hawassa.95% confidence interval margin of error of 4%. The final sample size was 553.

Stratified sampling techniques were used for selecting the study subjects. First of all healthcare workers were stratified in to private & government then further classified according to their type or level. Proportional numbers of health care workers were selected from each strata of health facility by simple random sampling technique.

### Data collection tools and procedures

Data were collected by structured self administered questionnaire which is adapted to fit with this research objective from [ILO/ICN/WHO/PSI] [14] after it is pretested & prepared in Amharic. The questionnaire was divided in to four parts. The first part was socio-demographic & occupational characteristics like gender, age, educational status, profession, type of facility, working hour, working section and marital status. The second, third & fourth section contains physical violence, verbal abuse & sexual harassment respectively with relevant related questions. Four environmental and occupational health professionals working in the city was used to collect the data from. Two environmental health professional from Gondar university student services were assigned to supervise the data collection process. Both data collectors and supervisors was given a one day training on aim of study procedures of collection & exercise it. The questionnaire was discussed thoroughly question by question. The study participants were made to fill the questionnaire in their respective health facility.

### Data processing and analysis

All the questionnaires were checked manually, coded and entered in to EPI info version 7.1.5.2 and exported to SPSS version 20 software for analysis of potentially explanatory variables. Descriptive analyses were performed to describe variables using summery measure, frequencies, figures & tables. 12 month WPV was evaluated by running bivirate logistic regression. Then variables with the *P*-value ≤0.2 analyzed in multivariable regression. The degree of association between dependant & independent variables was assessed using odds ratios within 95% confidence interval *p*-value ≤0.05. workplace violence is ascertained when the study respondents experienced at least one type of workplace violence (i.e. physical violence, verbal abuse or sexual harassment) in circumstances related to their work in the past 12 months.

## Results

### Socio demographic characteristics of respondents

The response rate was 96.02% (*N* = 531).Among respondents 361(68.0%) were from government & private hospitals and the rest were from private clinics and health centres. In addition 255(48.0%) were males and 276(52.0%) were females. The median age was 27, IQR = 7 years with the range of 20 to 56. Majority 289(54.4%) of healthcare workers are between the age group of 26–35 years. Among total participants majorities 415(78.2%) are belong to government health facilities while the rest work at private facilities116 (21.8%).In respect to educational status 405(76.3%) of HCWs have degree & above qualifications in their professions (Table 1).

**Table 1:** Socio demographic characteristics of health care workers working at health facility at Gondar city administration, March 2016(*n* = 531)

Variables	Frequency	Percentage
Sex		
Male	255	48.0
Female	276	52.0
Age		
≤ 25	76	14.3
26–35	289	54.4
≥ 36	166	31.3
Religion		
Orthodox	454	85.5
Muslim	56	10.5
Others	21	4.0
Educational status		
Diploma	126	23.7
Degree	361	68.0
Masters	31	5.8
Specialty	13	2.4
Marital status		
Married	245	46.1
Divorced	10	1.9
Single	276	52
Profession		
Nurse/midwife	339	63.8
HO	34	6.4
Pharmacy/laboratory	111	20.9
Other HCWs	24	4.5
GP	23	4.3
Facility ownership		
Private	116	21.8
Government	415	78.2

NB: Others: Protestant/catholic/Adventist

### Prevalence of workplace violence

More than half (58.2%) [95% CI: (53.7, 62.3)] of health care workers experienced at least one of manifestation of workplace violence (physical, verbal & sexual) in the past 12 months. Health care workers mostly encountered verbal abuse 282(53.1%) followed by physical attack 117(22.0%) and 38(7.2%) sexual harassment. Among all one third (33.0%) of health care workers were a victim of two forms of workplace violence of the study while only 4.2% of participant reported they were experience all forms. Over one third (37.9%) had witnessed physical violence on colleagues on their working environment. Females are most exposed in all forms of workplace violence: verbal abuse 161(57.1%), physical attack 69(59.0%) & sexual harassment 38(100%) than men.

### Workplace characteristics of healthcare workers

Two third 363(68.4%) of respondents reveal that unavailability of workplace violence reporting procedures in their health facility. Inpatient departments are places where one third 168(31.6%) of the health care workers spent their time in the health facility. More than half of all health care workers have short experiences of less than six years (Table 2).

**Table 2:** Organizational and workplace characteristics of healthcare workers working at health facilities of Gondar city administration, March 2016, (*n* = 531)

Variables	Frequency	Percentage
Facility type		
Hospital	361	68.0
Health center	92	17.3
Private clinics	78	14.7
Violence Reporting procedure		
Available	168	31.6
Unavailable	363	68.4
Department		
Inpatient departments	188	35.4
Pharmacy/laboratory	108	20.3
Emergency departments	46	8.7
Other departments	25	4.7
OPD	164	30.9
Experiences(in years)		
1–5	308	58.1
6–10	151	28.4
11–15	15	2.8
16–38	57	10.7
Job position		
Staff/service provider	484	91.2
Ward/clinic head	40	7.5
Coordinator	7	1.3
Shift work		
Yes	336	63.3
No	195	36.7

### Associations between exposure to types of violence and organizational and workplace characteristics

Higher risk of physical violence was related to working at shift, in inpatient department, govermet facilities and having lower years of experience. A total of 54.7% of health care workers with fewer than 5 years of experience reported physical violence, which decrease & increase with increase in experience. Physical violence was mostly reported in government workers (86.3%) compared with privately owned facilities. There is no any association is observed between all forms of violence in relation to job position & availability of reporting procedure. Verbal abuse showed stronger relationship similar to physical violence with stronger risk in facility ownership. Sexual harassment demonstrated lower relationship with organizational & workplace characteristics, which is limited with working department & ownership of the facility (Table 3).

**Table 3:** Organizational and workplace characteristics of healthcare workers working at health facilities of Gondar city administration with type of violence, March 2016, (*n* = 531)

Variable	Physical violence		Verbal abuse		Sexual harassment	
	yes	no	yes	no	yes	no
	*n* (%)	*n* (%)	*n* (%)	*n* (%)	*n* (%)	*n* (%)
Job position						
Staff/service provider	107(22.1)	377(77.9)	231(47.7)	253(52.3)	37(7.6)	447(92.4)
Ward/clinic head	9(22.5)	31(77.5)	24(60.0)	16(40.0)	1(2.5)	39(97.5)
Coordinator	1(14.3)	6(85.7)	5(71.4)	2(28.6)	0(0.0%	7(100.0)
Shift work						
Yes	96(28.6)**	240(71.4)	209 (62.2)**	127 (37.8)	29 (8.6)	307 (91.4)
No	21(10.8)	174(89.2)	73 (37.4)	122 (62.6)	9 (4.6)	186 (95.4)
Experiences(in years)						
1–5	225(54.3)*	83(70.9)	184(65.2)*	124(49.8)	37(97.4)	271(55.0)
6–10	130(31.4)	21(17.9)	67(23.8)	84(33.7)	0(0.0)	151(30.6)
11–15	14(3.4)	1(0.9)	6(2.1)	9(3.6)	0(0.0)	15(3.0)
16–38	45(10.9)	12(10.3)	25(8.9)	32(12.9)	1(2.6)	56(11.4)
Department						
Inpatient departments	64(54.7)**	124(30.0)	119(42.2)**	69(27.7)	20(52.6)*	168(34.1)
Pharmacy/laboratory	6(5.1)	102(24.6)	53(18.8)	55(22.1)	3(7.9)	105(21.3)
Emergency departments	22(18.8)	24(5.8)	34(12.1)	12(4.8)	6(15.8)	40(8.1)
Other departments	3(2.6)	22(5.3)	7(2.5)	18(7.2)	2(5.3)	23(4.7)
OPD	22(18.8)	142(34.3)	69(24.5)	95(38.2)	7(18.4)	157(31.8)
Ownership						
Private	16(13.7)*	100(24.2)	44(15.6) **	72(28.9)	3(7.9) *	113(22.9)
Government	101(86.3)	314(75.8)	238(84.4)	177(71.1)	35(92.1)	180(77.1)
Violence Reporting procedure						
Available	37(31.6)	131(31.6)	44(15.6)	72(28.9)	10(26.3)	158(32.0)
Unavailable	80(68.4)	283(68.4)	238(84.4)	137(71.1)	28(73.7)	335(68.0)

NB: statically significant at * = *p* < 0.05, ** = *p* ≤ 0.0001

### Factors associated with workplace violence

In univarate analysis profession, level of facility, experience, department, age, Employment status, health facility ownership, shifts work becomes significantly associated with workplace violence. In fitting these variables in to multivariate analysis only, department, profession, shift work and experience remain significant.

Occupational setting of health care workers demonstrated that the odds of violence against health care workers were nearly about four times higher among emergency department workers than those served in outpatient department (AOR = 3.99, 95% CI: (1.49,10.73)). Working at shifts revealed that it exposed to violence two times compared to those who worked at day shifts (AOR = 1.98, 95% CI*:* (1.28,3.03)).Health care providers of 1–5 years of experiences are three times at risk of encountering violence at work in contrast to 16+ year served colleagues (AOR = 3.09, 95% CI: (1.20,7.98)). Working as nurse & midwife in the health care facilities is four times more likely to experience violence than the general practitioners (AOR = 4.06, 95% CI: (1.20,13.74)) (Table 4).

**Table 4:** Univariate & multivariate logistic regression of factors associated with workplace violence among health care workers working at health facilities in Gondar, March 2016(*n* = 531)

Variables	Workplace violence		COR(95% CI)	AOR(95% CI)
	Yes	no		
Working department				
Emergency	40	6	7.71(3.10,19.20)***	3.99(1.49,10.73)**
Inpatient	131	57	2.66(1.71,4.11)	1.41(0.78,2.55)
Other^a^	8	17	0.54(0.22,1.33)	0.42(0.16,1.11)
Pharmacy/laboratory	54	54	1.15(0.71,1.88)	0.91(0.24,3.47)
OPD	76	88	1	1
Shift work				
Yes	227	109	2.87(1.99,4.13)***	1.98(1.28,3.03)**
No	82	113	1	1
Years of experiences				
1–5	206	102	2.40(1.35,4.26)**	3.09(1.20,7.98)*
6–10	71	80	1.05(0.57,1.95)	1.38(0.55,3.51)
11–15	6	9	0.79(0.25,2.52)	1.47(0.36,6.01)
16+	26	31	1	1
Occupation				
Nurse/ Midwife	221	118	6.74(2.44,18.62)***	4.06(1.20,13.74)*
HO	13	21	2.23(0.66,7.46)	2.49(0.61,10.06)
Pharmacist/Laboratory	56	55	3.66(1.27,10.56)	3.63(0.60,21.88)
Other^b^	14	10	5.04(1.40,18.14)	4.06(0.91,18.11)
GP	5	18	1	1
Ownership				
Private	49	67	1	1
Government	260	155	2.29(1.50,3.49)***	1.22(0.56,2.66)
Type of facility				
Hospitals	231	130	3.35(2.00,5.61)***	1.20(0.48,3.03)
Health center	51	41	2.35(1.26,4.37)	1.23(0.44,3.43)
Private clinics	27	51	1	1
Age(in years)				
≤ 25	36	40	0.55(0.32,0.95)*	1.69(0.69,4.14)
26–35	170	119	0.87(0.59,1.29)	1.38(0.86,2.25)
≥ 36	103	63	1	1
Employment				
Full timer	296	201	1	1
Contract	13	21	0.42(0.20,0.85)*	1.10(0.42,2.87)

NB: statically significant at * = *p* < 0.05, ** = *p* ≤ 0.006, *** = *p* ≤ 0.0001

^a^liaison, central supply, physiotherapy, anaesthesia, x-ray, card room, triage

^b^physiotherapist, anthesist, optometrists, psychiatrists, radiographers

## Discussion

To the best of our knowledge this research is the first comprehensive research of workplace violence on health care both in profession & type of health facilities covered in Ethiopia. Being nurse/midwife by profession, working in emergency department, shift work and having short experiences are significantly associated with workplace violence.

The study finds out 58.2% of respondents encounter WPV. This is higher than what is reported by WHO. The overall 12 month prevalence of workplace violence among healthcare workers is in line with study findings in South Africa (61.9) [3], Thailand (54.1) [15] and Turkey(57.5%) [16]. This may be due to the methodological similarities employed in the studies. And it is lower than study in Nigeria (69.4) [17] and Oromiya, Ethiopia (88.0) [18] as it may be the fact that both studies doesn’t include private sectors which relatively have low prevalence than government counterparts. Even the Ethiopian study is only on hospitals & nurses in which known for their higher prevalence of workplace violence.

Workings in emergency departments have positive association with workplace violence. Those who work in clinical setting of emergency are four times exposed to workplace violence than OPD workers. An emergency working setting is where peoples are come in panic, with serious injuries that make them to be aggressive at health care providers. This is a place where life threatening health conditions and death make visitors & patient relatives to be violent. All these fuelled by nervousness of HCWs which attributed to high workload & stress. This finding is similar with study conducted in Hawassa, Ethiopia [13]. Similar emergency service delivery system, violence handling & security condition may account for the similarity of results. In addition despite of target population difference those who work in emergency departments mostly are nurses. A more higher risk reported from Italian [19] study on both physical violence & threats. This disagreement happens since healthcare workers from developed nation will report incidents more frankly & correctly as their system responds proactively for employees safety.

Shift work appears to be an exacerbating factor for the encountering of workplace violence among health care workers. Those working at shifts are more likely to experience workplace violence than their colleagues of day shift. This finding is supported study from China [2]. Working in shift implies low level of security in the institution, fewer staff in the department and decreased work performance between staffs initiate conditions favourable for violence. Even limited or no presence of hospital administration also can be attributed. While a study from Turkey [20] shows lower association than our research. This might be due that higher workplace violence prevention interventions are provided in such developed country than this study setting.

Year of experience in health facilities have positive association with the occurrence of workplace violence. Those who have less than 6 years of experience 3 fold more likely victimized by violence than their seniors with more than 16 years of experience in the health care facilities. This may be health care workers with short experience and mostly young are lacking the skills of managing violent conditions which can be acquired through experiences. The result is less than a study in Hawassa [13]. The difference may be due the difference in study subjects by profession. As Hawassa study is only on the nurses, who are deemed the most vulnerable while our study comprises all health professionals. The Congolese [21] study come up with a nearly no association results. The inconsistency can be by difference in sample size as the Congolese one is a nationwide study & it doesn’t includes violence arising from co-workers which leads to a normality across all experience categories.

Practicing a specific profession is a significant factor that exhibits visible association with workplace violence. Being a nurse or midwife had increased encountering workplace 4 times than working as physician. This is supported by research from Saudi [22]. This can be reason out since nurses are the front liners in giving service in health facilities in which patients & patient’s relatives spent more of their health facility times with them. Lower risk is reported from Brazil [23] & Serbia [24]. This observable difference may be the difference of healthcare system in which professionals exposed to patients & other potential sources of violence. In addition the proportion of nurses or midwifes & physicians involved in these studies are not as large as this study involves which decrease the risk of exposure.

In this study Respondents rated long waiting time for the service and lack of security condition as the primary causes that facilitate occurrence of workplace violence. This claim of HCWs supported by researches from the Middle East countries [22, 25].when patient/client wait for long time to get service they become irritated & dissatisfied which results to quarrelling with HCWs and even assaulting verbally & physically. Addressing long waiting time is also matter of improving the quality of service that ministry of health strives.

The study clearly shows that policy & working strategies should steer towards reducing factors aggravating workplace violence: such as log waiting time. In addition result related to absence of violence reporting procedure significantly related to having effective prevention of workplace violence.

The study will come with possible limitations like Recall bias which emanates as respondents expected to remember the past 12 month exposure. Having wider study subject coverage by profession & Inclusion of both government and private facilities will be considered as strength since it will give a picture of all health care workers.

## Conclusions

Workplace violence appears to be major occupational hazard & public health problem despite it is neglected both by victims and health facilities. Short experiences, working in emergency department, shift work & being nurse/midwife has positive association with workplace violence.

## Abbreviations

ART: Anti retroviral therapy
EPI-info: Epidemiological Information
GP: General Practitioner
HCW: Health Care Worker
ICN: International Council of Nurses
ICU: Intensive care unit
ILO: International Labor Organization
IQR: Inter quartile range
MCH: Maternal and child health
OPD: Outpatient department
PSI: Public Service International
SPSS: Statistical Package for the Social Sciences
TB: Tuberculosis
WHO: World Health Organization
WPV: Workplace Violence
